# Parametric Loop Division for 3D Localization in Wireless Sensor Networks

**DOI:** 10.3390/s17071697

**Published:** 2017-07-24

**Authors:** Tanveer Ahmad, Xue Jun Li, Boon-Chong Seet

**Affiliations:** Department of Electrical and Electronic Engineering, Auckland University of Technology, Auckland 1010, New Zealand; tahmad@aut.ac.nz (T.A.); xuli@aut.ac.nz (X.J.L.)

**Keywords:** parametric loop division, triangulation, centroid, range-free localization, wireless sensor networks

## Abstract

Localization in Wireless Sensor Networks (WSNs) has been an active topic for more than two decades. A variety of algorithms were proposed to improve the localization accuracy. However, they are either limited to two-dimensional (2D) space, or require specific sensor deployment for proper operations. In this paper, we proposed a three-dimensional (3D) localization scheme for WSNs based on the well-known parametric Loop division (PLD) algorithm. The proposed scheme localizes a sensor node in a region bounded by a network of anchor nodes. By iteratively shrinking that region towards its center point, the proposed scheme provides better localization accuracy as compared to existing schemes. Furthermore, it is cost-effective and independent of environmental irregularity. We provide an analytical framework for the proposed scheme and find its lower bound accuracy. Simulation results shows that the proposed algorithm provides an average localization accuracy of 0.89 m with a standard deviation of 1.2 m.

## 1. Introduction

Recent advancements in wireless communication and electronic systems make wireless sensor networks (WSNs) as a prominent asset of Internet of Things (IoT). A WSN is composed of large number of sensor nodes that are densely deployed in a field. Each node is equipped with a central processor, several sensing modules, limited memory, and a transceiver [1]. WSN nodes are capable of processing information on pre-programmed routines, communicating with other nodes and sending the collected data to a sink node. The feasibility of fast deployment and low cost solution make WSNs promising for different applications, such as security surveillance, home automation, human interfacing and livestock farming.

Localization of sensor nodes is a challenging issue in WSNs. Localization systems are not only for location identification but also for routing, density control, tracking and a number of other communication network applications, which integrate in many technologies of IoT. Localization systems can be classified as outdoor localization and indoor localization system. Global Positioning System (GPS) is the de facto standard for outdoor localization that provides global coverage and its precision up to 1–5 m. Although it is straightforward to assume that each sensor node have a GPS module or an additional ranging module for localization, this method is fairly limited due to the consideration of manufacturing cost and computational power of affordable sensor node [2]. Due to its strict requirement of line of sight (LoS), GPS is not suitable for indoor localization. Moreover, the GPS receivers are costly and consume more power as compared to sensor modules [3]. An indoor positioning system (IPS), locates objects or people inside a building using radio waves, magnetic fields, acoustic signals, or other sensory information collected by mobile devices. There are several commercial systems on the market, but there is no standard for an IPS system. Different techniques can be applied, which include distance measurement to nearby anchor nodes (nodes with known positions, e.g., Wi-Fi access points), magnetic positioning, dead reckoning. They either actively locate mobile devices and tags or provide ambient location or environmental context for devices to get sensed.

**Figure 1:**
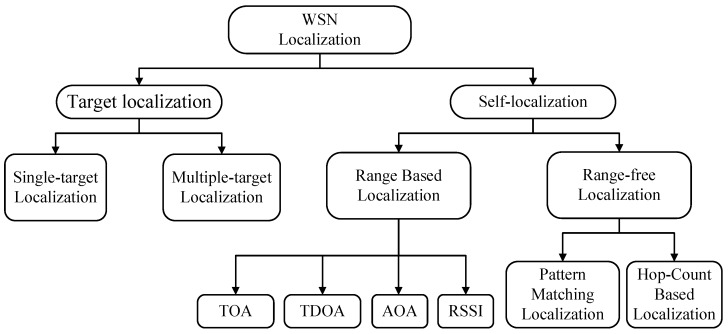
Classification of localization algorithms.

Localization techniques can be broadly classified as target localization and self-localization, where the former aims to locate the position of a transmitting node and the latter aims is to localize a node by itself using received signals from neighbor nodes as shown in Figure 1. Target localization requires distinctive activities to work properly [4]. Many localization algorithms have been proposed in different areas. Generally speaking, based on the type of information required for localization, self-localization algorithms can be divided in two categories: (1) range-based and (2) range-free, where range-based techniques use additional ranging modules or received signal strength indicator (RSSI) measurement to perform localization, and range-free techniques utilize the connectivity information between sensor nodes to coarsely localize sensor nodes. Although each method has its own merits and de-merits, its primary target is to estimate the position of sensor node while considering accuracy, power efficiency and complexity.

### 1.1. Range-Based Localization Techniques

Range-based localization techniques first estimate distance information using different methods, such as Time of Arrival (ToA) [5], Time Difference of Arrival (TDoA) [6], Angle of Arrival (AoA) [7] and other methods that are based on RSSI [8,9,10,11,12]. The location of a node is then found by using traditional triangulation, trilateration or maximum likelihood estimation methods [13,14]. In [15], the authors presented a one-dimensional scheme that used a ratio of signal strength instead of absolute signal strength to perform localization. Several approaches only considered that closer nodes obtained higher RSSI values [16]. However, it is only reliable when coordinates are known from the RSSI database. Due to the non-line of sight (NLOS) and multipath fading, signal propagation model becomes complex and often causes large localization error [17,18,19]. All four methods stated above, except RSSI based techniques, provided a superior localization accuracy. However, they are not suitable for large-scale sensor networks due to expensive hardware required for ranging purpose.

### 1.2. Range-Free Localization Techniques

Range-free techniques adopt sensing features like wireless connectivity, localization event detection and beacon/anchor node proximity that leads to a low-cost solution, but at the expense of localization accuracy [20,21,22]. Fingerprinting based localization [23] is a typical example of range-free localization, accomplished in two phases—offline phase and online phase. In the offline phase, a database of the RSSI values from different access points at each reference location for the target environment is built. In the online phase, the node position is estimated by a localization algorithm using the collected RSSI at that particular position and the RSSI database (known as RSSI fingerprints) from the offline phase. In this category, several techniques, such as the ray tracing model [24], support vector machine [25], data mining techniques [26], probabilistic [27], and others based on Kalman filtering [28] have been designed to collect online RSSI samples to be stored in the offline database. Notice that fingerprinting may provide false results due to indoor multipath effects, such as reflection, diffraction and scattering.

The range-free approach becomes more useful in WSN localization due to low-cost, reasonable accuracy and low power consumption [29,30,31]. Ad hoc Positioning System (APS) [32], Multidimensional scaling map (MDS-MAP) [33] and Approximate point in triangulation (APIT) [31] are typical range-free technique for WSN localization. However, range-free localization depends on the spatial distribution of anchor nodes that may vary with different environmental conditions.

Indoor localization can be applied to enable a variety of location-based services in commercial or residential environments. Consequently, different technologies can be chosen according to particular system requirements. Table 1 shows the sensor technologies along with their coverage and measured accuracy.

Zigbee is a promising technology for low rate wireless personal area network (WPAN), and it features low power consumption and low data throughput. Its communication range is 100 m and 30 m for free space and indoor environment, respectively. The distance estimation between two Zigbee nodes is usually carried out through manipulating RSSI values. Since overall system cost is a main issue in industrial and home wireless applications, a highly integrated single-chip approach is the preferred solution of semiconductor manufacturers developing IEEE 802.15.4 compliant transceivers. The IEEE standard is the significant factor in determining the RF architecture and topology of ZigBee enabled transceivers. The ZigBee group was organized to define and set the typical solutions for these layers for star, mesh, and cluster tree topologies. Therefore, there is significant need for some automated process to help discover, identify, and locate the nodes within an indoor facility after the installation takes place. This motivates us to propose a localization algorithm that is designed for Zigbee based WSNs.

The rest of the paper is organized as follows. Section 2 discusses the state of the art work done in WSN localization. Section 3 presents the proposed localization scheme for WSN localization, followed by its analytical framework in Section 4. Section 5 discusses the simulation results, computational complexity and lower bound of the proposed localization algorithm. Section 6 concludes the paper with possible future work.

## 2. Related Work

Design of an IPS requires thorough analysis of specific application descriptions and user requirements in order to justify the research and development in the field. In particular, a localization algorithm should be (1) accurate–maintains low localization error; (2) robust–maintains its performance in different environments; (3) energy efficient–in communication and computation for battery-operated sensor nodes; (4) reliable–tolerant of node failures. Localization algorithms are measured in terms of localization accuracy and power consumption.

APS was proposed in [32]. Under APS, multilateration was initially impossible because no sensor node can receive beacons from at least three anchors. Localization was performed based on a hybrid method combining distance vector like propagation and GPS triangulations. Immediate neighbors of those anchors were used to calculate the distance between anchors and their neighbors. Similarly, location estimation was propagated from the anchors towards the center of the network. APS is distributed and does not require special infrastructure. Furthermore, it provides global coordinates with good accuracy.

Multi-dimensional scaling (MDS) based algorithm was proposed in [33]. MDS was from mathematical psychology, which provides a method to display the structure of distance-link data as a geometrical picture. The proposed MDS-MAP scheme had three steps: (1) Estimation of distance between each possible pair of nodes; (2) Derivation of node localization using MDS to fit those estimated distances; (3) Normalization of the resulting coordinates using known information of anchor nodes. MDS-MAP could generate a relative map of nodes without any anchor node. With three or more anchor nodes, the absolute coordinates of nodes can be estimated.

APIT technique proposed in was based on Point-in-Triangle Test (PIT) [31] , under which a target node chooses three beacon nodes and then tests whether it is inside the triangle or not by connecting three beacon nodes. APIT algorithm has four steps: (1) Reference exchange; (2) PIT Test; (3) APIT aggregation; and (4) Centre of gravity Calculation (centroid localization). Simulation results showed that APIT outperformed other existing techniques and provided better results with lower communication overhead under irregular radio patterns and random node placement.

Mostly works focus on 2D localization. That is why, in this paper, we proposed a 3D localization scheme based on parametric Loop division (PLD) to improve localization accuracy, to minimize the computational load and to mitigate the dependence of anchor node deployment. Loop subdivision algorithm is widely used for its simple rules, excellent continuity, and its triangular controllable meshes [34]. Loop subdivision is a surface split approach that is based on 3-order B-spline. With the help of control vertices each parametric node is calculated on the earth space with in step size. Triangulation mesh is used for pre localized point. However, it is different from APIT that gets location information from overlapping triangles.

## 3. Proposed PLD Localization

The key notations used in the proposed PLD scheme are summarized in Table 2.

### 3.1. Basic Principle of PLD Algorithm

The key idea of the PLD algorithm is to find an actual localization volume in 3D space and estimate the actual position of a node. Ref. [34] provides an example of a subdivision, where 3D images are generated from triangular subdivision method. In each step, it sub-divides parts of a triangle with the addition of extraordinary nodes in its control ring matrix. Three nodes in a given network can form a triangle.

We select the nearest node as a reference point and produce new parametric points with the help of those extraordinary nodes. This work involves the development of novel solution for ZigBee based localization and utilizes the knowledge of fixed node positions to calibrate nodes with unknown positions. This will allow the positioning systems to adapt themselves in a changing environment, thereby increasing accuracy and reliability.

**Figure 2:**
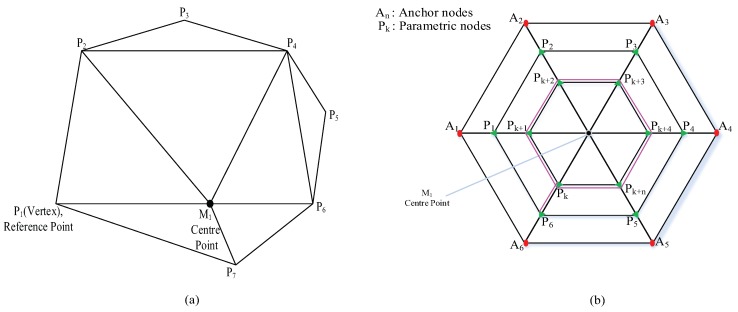
(**a**) Triangulation of Parametric nodes distribution from control vertices; (**b**) Parametric points calculation in Loop division.

New parametric points are produced with the help of those reference points. Inner node distribution of parametric node using Loop division is found in triangulation form as shown in Figure 2a.

PLD is applicable for localizing a WSN node using both uniform and random distribution of anchor nodes. PLD produces different iterations and each iteration may have a number of unknown nodes in a high volume of anchor node distribution in 3D space. Furthermore, each PLD network is capable of calculating its actual number of nodes for localization. The parameterization of 3D space through PLD algorithm produces parametric node, where each node has information about sum of received power from all anchor node of network. By using the parametric node, each iterative step in PLD is capable of producing a similar triangle with respective anchor nodes.

At each parametric point, sum of RSS from all anchor nodes is checked against a pre-defined threshold. If it is smaller than the threshold value, the corresponding parametric point will be added to the storage matrix. Otherwise, if it is larger than the threshold value we neglect those points at this step. If all side threshold points are found, the loop will be terminated. And mid-point position is shifted up and down by step size Δ. After recording the upward and downward step size we pick up storage matrix and calculate the actual localizing volume. The position of a sensor node is estimated through centroid method, and the localization error can be calculated.

As shown in Figure 2b, let $\Delta M_{1}A_{1}A_{2}$ be our chosen triangle. After the first iteration with the basis function, a similar triangle is formed as $\Delta M_{1}P_{1}P_{2}$. Continuous parametrization in a Loop produces similar triangulation structures. The shifting from one network to another is done under the basis function parameters, which shrinks the volume of triangulation structure. The parametric points $P_{1}$ and $P_{2}$ are produced through parametric equation and re-calculate the mid-point $M_{1}$ as previously defined in [35]. This small variation in center point further helps in correcting random anchor node distribution. For a proper distribution system, (A+1)th anchor node is followed by *A*th anchor nodes in a PLD network.

### 3.2. Problem Formulation and Assumptions

Consider a non overlapped network $K=k_{1},k_{2},\ldots k_{n}$ with volume $V=v_{1},v_{2},\ldots v_{n}$. Assume that a WSN with *N* sensor nodes and *A* anchor nodes are randomly deployed in a sensing field. Each sensor node maintains a set of parameters as:

(1) $$N=\{N_{i}\left(x_{i},y_{i},z_{i}\right),A_{i}\left(x_{i},y_{i},z_{i}\right),D_{N\to N},D_{N\to A}\},\;i=1,2,3,\ldots ,n$$

where $x_{i},y_{i},z_{i}$ are the coordinates of the *i*th node. Similarly, each anchor node maintains a set of parameters as:

(2) $$A=\{N_{i}\left(x_{i},y_{i},z_{i}\right),A_{i}\left(x_{i},y_{i},z_{i}\right),D_{A\to N},D_{A\to A}\},\;i=1,2,3,\ldots ,n$$

As there are *N* sensor nodes and *A* anchor nodes, the node position in a 3D space can be denoted as

(3) $$n_{i}={\left(x_{i},y_{i},z_{i}\right)}^{T}for\;i=1,2,\ldots N+A$$

Assume each PLD network has φ number of nodes with unknown positions and η anchor nodes, which results in k × φ and k × η nodes in the network. The value of ρ is a constant, and it should be greater than 4 for proper parametric Loop formation. The Eculidian physical distance between two sensor nodes $n_{i}$ and $n_{j}$ is $d_{\mathrm{ij}}=\sqrt{{\left(n_{i}-n_{j}\right)}^{2}}$. Furthermore, in each PLD network, the proximity information between sensor nodes is $P_{ij}\in \beta_{k}=\left\{1,2,\ldots \varphi +\eta \right\}$, anchor nodes $n_{\eta }$, geographic physical distance $d_{ij}$ estimates the position of $n_{\varphi }$ where $\varphi \in \left\{\eta +1,\eta +2,\ldots \eta +\varphi \right\}$.

Anchor nodes are deployed with known positions. Consider a 3D WSN with *n* small PLD networks, if there is no repetition in anchor node positions, there will be (N/K) number of possible PLD networks. In addition, it satisfies:

(4) $$\left(\frac{N}{K}\right)\leq N_{p}\leq N$$

where $N_{p}$ represents the each PLD network. For convenience, the following terms are defined to facilitate the discussion of the proposed PLD algorithm.

***Anchor Node***: A node whose position is known with the help of any positioning device, such as GPS.

***Reference Node***: A node that selects other nodes to form a triangle is known as reference node.

***Ring Control Matrix***: The anchor node position vector that acts as a boundary of a network is known as ring control matrix.

***Step Size***: The distance between parametric nodes of each loop is step size.

***Working Boundary***: The difference of each anchor/parametric coordinates maximum and minimum value is our PLD working boundary.

### 3.3. Algorithm Design

#### 3.3.1. Network Size, Midpoint and Parametric Points

Let a set of anchor nodes with position vector (x,y,z) be $A=\{A_{1},A_{2},A_{3},\ldots A_{m}\}$, where m≥4. Each reference anchor node select another two nodes to form a parametric triangle. For proper operation, the PLD network size should be greater than 3. Computation of the midpoint of a link between two anchor nodes with the maximum distance is the first step in parametric node selection. Let ${\vec{A}}_{1}$ be a reference node, the total distance between the *K*th selected nodes is:

(5) $$|{\vec{D}}_{1k}|=\sum_{k=2}^{m}|{\vec{D}}_{Ak}|$$

By computing parametric factor, the control will be transferred to inner parametric points as shown in Figure 2b. Each anchor node will act as a control vertex in the first iteration, then the control is transferred to the next parametric point that forms a ring matrix by applying the following equations.

(6) $${\vec{P}}_{ik}=\frac{3}{8}\left({\vec{M}}_{1}+{\vec{A}}_{k}\right)+\frac{1}{8}\left({\vec{A}}_{k-1}+{\vec{A}}_{k+1}\right)$$

#### 3.3.2. Selection of Pre-Localized Nodes, Step Size and Storage Matrix

The next step is to check RSSIs from anchor nodes at each parametric point. In this paper, the RSS calculation follows

(7) $$RSSI=P_{T}-P_{L}+F_{D}$$

where $P_{T}$, $P_{L}$ and $F_{D}$ denotes the transmission power from an anchor node, the path loss and the fading, respectively. The upward increment and downward increment of the center point is obtained by addition and subtraction of step size over the working boundary. If the sum of RSS values are smaller than the threshold value, it is chosen as a pre-localized node and the iteration stops at this point. Spherical distance is calculated using the PLD coordinates $C_{k}$:

(8) $$C_{k}=\left[\begin{array}{ccc} x_{1,k} & y_{1,k} & z_{1,k}\\ x_{2,k} & y_{2,k} & z_{2,k}\\ \vdots & \vdots & \vdots \\ x_{i,k} & y_{i,k} & z_{i,k} \end{array}\right]$$

#### 3.3.3. Estimation of Node Position

Maximum and minimum values of each coordinate axis are found from (x,y,z) from the storage matrix. Then, product of the difference between the maximum and the minimum values on each coordinate axis is regarded as the localization volume, which is computed by

(9) $$V=\left(x_{\max}-x_{\min}\right)\left(y_{\max}-y_{\min}\right)\left(z_{\max}-z_{\min}\right)$$

To find localization points, we calculate the volume of pre-localized node boundary in Cartesian coordinate form and divide it by each unitary volume.

(10) $$V_{u}=\frac{V}{N}$$

where $V_{u}$ represents the unit volume. To find the position of an unknown node, centroid based methods are used on the volume of the pre-localized node by taking vector difference between the minimum and the maximum value of pre-localized coordinate boundary on each of localized nodes.

(11) $${\left(\hat{x},\hat{y},\hat{z}\right)}_{l_{i}}=\prod \left[V_{u},C_{k}\left(a_{j}\right)\right]+{\left(x,y,z\right)}_{min}$$

where $l_{i}$ is a pre-localized node. Algorithm 1 describes the proceedure of Loop division.
**Algorithm 1** Description of PLD Algorithm1:take a network size φ2:**for** i=1:K **do**3:    calculate mid-point of the *k*th network4:    take step size Δ5:    divide the minimum axis difference into equal φ parts6:    **for** $i_{differ}=1:min_{axis}$ **do**7:        **for** $icase=1:min_{axis}/\varphi$ **do**8:           **if** $i_{differ}\geq min_{axis}/2$ **then**9:               accept positive step size10:               minpoint=midpoint+Δ11:               calculate the pre-localized points using Algorithm 212:           **else**13:               accept negative step size14:               midpoint=midpoint returning to old midpoint15:               minpoint=midpoint−Δ16:               calculate the pre-localized points using Algorithm 217:           **end if**18:        **end for**19:    **end for**20:**end for**21:find out each axis maximum and minimum points from the storage matrix22:calculate the volume of localization23:calculate the φ with the help of unit sensing volume.24:divide the storage pre-localized points to η25:**for** $i_{loc}=1:\eta$ **do**
26:    find a minimum and maximum coordinates from cluster of pre-localized points27:    calculate difference between minimum and maximum points28:    calculate the sensor position by adding difference and minimum co-ordinate of cluster29:**end for**


These pre-localized nodes are calculated through Algorithm 2.
**Algorithm 2** Calculation of pre-localized nodes1:**for** i=1:η+1 **do**2:    **for** j=1:η **do**3:        calculate parametric points4:        calculate distance between parametric points and each anchor nodes5:        calculate the sum of RSS from each anchor nodes6:        **if** sum(RSS)≤RSS(threshold) **then**7:           take a first parametric point corresponding to each anchor nodes8:           break9:        **else**10:           nodes with least sum of RSSI considered as pre-localized. Stored them in a matrix11:        **end if**12:    **end for**13:**end for**


Finally, we can calculate the localization error. Note that PLD uses triangulation meshes to compute parametric points, thus it is different from APIT that obtains location information from overlapping triangles.

As compared to existing localization algorithms, PLD has the following advantages: (1) PLD can achieve 100% network coverage by parametric Loop division under volume of pre-localized point. Settlement of step size helps PLD to work in a given boundary. On the contrary, we noticed that the performance of APIT degrades as communication range increases, and its network coverage does not reach 100%. Similarly, DV-Hop algorithm has the same issue. Moreover, the use of triangle meshs in PLD instead of overlapped triangle in APIT can overcome the communication cost and coverage problem. (2) Nodes distributions in PLD network are independent from connectivity, angle and other information that were pre-requisites for other localization techniques.

However, PLD is not perfect. The accuracy for PLD is dependent on the number of anchor nodes. Moreover, if anchor nodes are not deployed homogenously, some nodes may be located far from the mid-point. It happens if at least one of the anchor nodes is far from others in the outer boundary. Then, the step size of PLD falls far from that anchor node. Consequently, standard deviation of localization error increases, which indicates that the data points are spreaded out over a wider range of RSS values.

## 4. Analysis and Discussions

This section presents the robustness of PLD for applications with different anchor deployments. The location of parametric node lies on the selected polyhedron within working boundary of networks.

### 4.1. Calculation of Initial Center Points and Working Boundary

Let $A=\{{\vec{A}}_{1},{\vec{A}}_{2},{\vec{A}}_{3},\ldots ,{\vec{A}}_{m}\}$ be a set of anchor nodes in our localizing PLD network with reference anchor node ${\vec{A}}_{i}$. The Euclidean distance matrix between the reference anchor node ${\vec{A}}_{i}$ to an anchor node ${\vec{A}}_{j}$ is given by

(12) $$|D_{ij}|=\sqrt{{\left(X_{i}-X_{j}\right)}^{2}+{\left(Y_{i}-Y_{j}\right)}^{2}+{\left(Z_{i}-Z_{j}\right)}^{2}}$$

For the reference anchor node ${\vec{A}}_{i}$, the selection of another anchor node for midpoint calculation in a PLD network is determine by

(13) $${\vec{A}}_{k}=\underset{{\vec{A}}_{j}\in A}{\arg}\max\left|D_{ij}\right|$$

The mid-point of a PLD network is calculated as:

(14) $${\vec{M}}_{i}=\frac{1}{2}\left\{{\vec{A}}_{i}+{\vec{A}}_{k}\right\}$$

If anchor nodes are randomly deployed, the center points will result in more deviation as compared to regularly deployed case.

**Lemma** **1.** For regular distribution of anchor nodes, the midpoint of a PLD network is its centroid.

In regular distribution, PLD makes a regular shape in a 3D working boundary. The regular shape 3D object has diagonal of equal length where an intersection of all diagonals lies in the same place known as centroid points or center of mass point. The working boundary is calculated by:

(15) $$\xi =|f_{max}\left(x_{k},y_{k},z_{k}\right)-f_{min}\left(x_{k},y_{k},z_{k}\right)|$$

### 4.2. Center Points and Parametric Points Calculation

Let $M_{i}$ be the middle point of our working region. For *K*th anchor node PLD network, it stores initial control vertices in k×3 matrix. Generation of new points from existing points is based on a well-known theory in computer graphics [36], that gives the advantage of taking close location as extraordinary nodes and producing new parametric points with the help of those extraordinary nodes. The dimension of extraordinary nodes matrix is (K+1)×3 in a PLD network. The extraordinary node matrix in the first step is calculated as:

(16) $$B=\left[\begin{array}{ccccc} x_{M_{i}} & x_{A_{1}} & x_{A_{2}} & \ldots & x_{A_{k}}\\ y_{M_{i}} & y_{A_{1}} & y_{A_{2}} & \ldots & y_{A_{k}}\\ z_{M_{i}} & z_{A_{1}} & z_{A_{2}} & \ldots & z_{A_{k}} \end{array}\right]$$

The parametric points are generated using Equation (6). In addition, it has less effect from consecutive upper and lower anchor nodes. Due to static anchor nodes, ${\vec{P}}_{ik}$ mainly depends on center point of working boundary. Center point is dependent on both parametric factor and step size. Each new center point or midpoint has the effect described in Lemma 2.

**Lemma** **2.** If anchor nodes are regularly distributed, parametric factor becomes constant. Center point from the first step The first step center point and further step center point lies at the same point.

**Figure 3:**
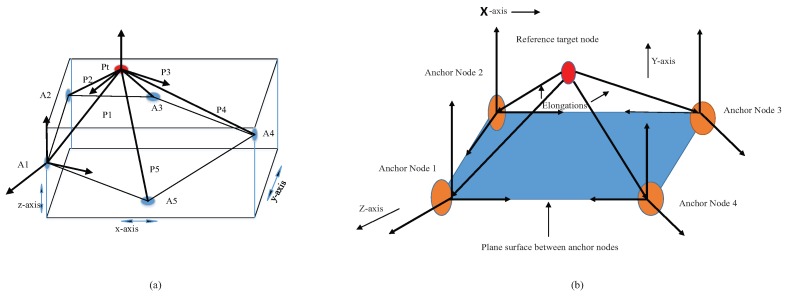
Triangulation and midpoint calculations in a parametric Loop division (PLD) network.

Equation (6) can generate parametric points within the working boundary. By taking advantage of choosing one point in each step of PLD, the adverse effect of irregular anchor node distribution is managed whose detailed derivation is shown in Appendix A. The anchor node distribution in a ring structure is shown in Figure 3.

**Lemma** **3.** The center points of a PLD network will shift due to changed parametric factor.

The parametric factor’s value depends on the angle that is made by center points between two anchor nodes. *K* anchor node makes *k* number of the same and different angles, which is dependent on the nodes distribution. In the regular distribution of anchor node, all angle are acute angle except k=3 and k=4. If random anchor node distribution occurs, then some angles become obtuse. In the whole of the process, we take corresponding angle value. The sum of all angles is equal to 360°. If anchor nodes is equal to or more than four, then parametric factor varies from 0.765 to 0.516, and angle ranges from 90 to 0°. The first element is obtained by assuming constant distribution and the second one is derived from the average value of different parametric factor. Shifting of the center point is detail derived in Appendix B.

### 4.3. Movement of Mid-Points in PLD Network

The proper distribution of anchor nodes produces midpoints in the exact center of PLD network. However, there is no need of exact location of center points in PLD because midpoint always varies within the working boundary. The step size Δ on each axis coordinate gives uniform and random movement of medium points. These variations are calculated by the following equations.

(17) $$M_{1}=\left\{\left(M_{x}\pm \Delta \right),\left(M_{y}\pm \Delta \right),\left(M_{z}\pm \Delta \right)\right\}$$

(18) $$N_{mov}=\frac{\xi }{\Delta }$$

where $N_{mov}$ represents the change in sensor nodes position.

**Lemma** **4.** *The considerable shift of middle point does not affect the parametric factor* $\alpha_{k}$.

**Figure 4:**
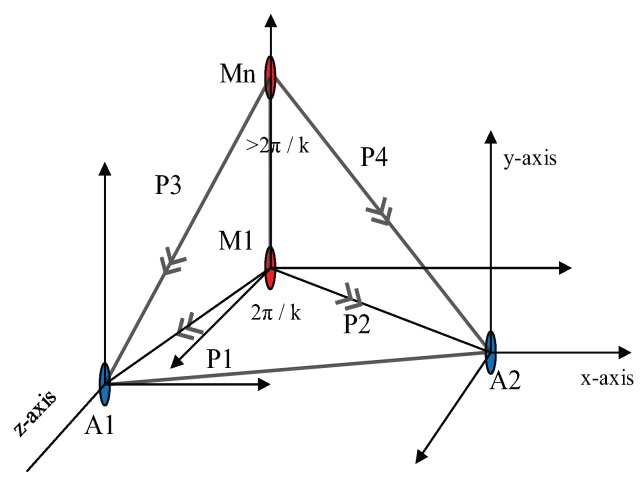
Effect on parameterization with various parametric factors.

The shifting of middle points upward and downward is considerable in our working boundary. By shifting midpoints in an upward direction as shown in Figure 4, then each angle of the PLD network changed. The deviation of parametric factor calculated by:

(19) $$\sigma_{\alpha_{k}}=\frac{3}{16}\left(\cos\theta_{max}-\cos\theta_{min}\right)+\frac{1}{8}\left(\cos^{2}\theta_{max}-\cos^{2}\theta_{min}\right)$$

In an experimental study, it is observed that the change in midpoint does not affect the performance. Suppose, with the six anchor nodes and there is a change of 5° upwards and 5° downwards in midpoint angle, it produces negligible change in the parametric factor. As finding the exact location of midpoint is not necessary for the PLD network, the effect of the change in Cosine angle is also not important. Therefore, assuming that a PLD network in WSN is independent of considerable angle variation on a parametric factor.

### 4.4. Computation of Pre-Localized Nodes

The PLD model finds out a number of parametric nodes in each step by parameterization near to extraordinary nodes. The sum of RSSI is checked at each parametric point and the distance between each parametric point and anchor node position is utilized for RSSI power calculation. From equation of RSSI the path loss is:

(20) $$PL\left(d\right)\left[dB\right]=PL_{F}\left(d_{o}\right)+10n\mathrm{lg}\left(\frac{d}{d_{0}}\right)$$

By the central limit theorem, RSSI can be represented by Gaussian random complex variable and the Rayleigh PDF is given by:

(21) $$f_{X}\left(x\right)=\frac{x}{\sigma^{2}}.e^{-\frac{x^{2}}{2\sigma^{2}}}$$

The RSSI values and distance have the following empirical relationship as verified in [37]

(22) RSSI(dB)=−23.28×lg[d(m)]−2.4225

The sum of RSSI at each node is:

(23) $$\sum RSSI=\sum_{k=1}^{K}{\overset{´}{D}}_{RSSI}$$

$${\overset{´}{D}}_{RSSI}=|{\vec{P}}_{ik}-{\vec{A}}_{ik}|$$

Finally, the RSSI valuse are being stored in a matrix by following relation:

(24) $$f\left(P_{RSSI}\right)=\left\{\begin{array}{cc} Preloc_{cord} & (P_{RSSI})\leq threshold\\ {}* & otherwise \end{array}\right\}$$

### 4.5. Storage Reduction Factor and Actual Node Calculation

The regular distribution of anchor nodes lies in triangulation vertex with step size Δ. But anchor nodes practically have a random distribution in 3D space, therefore introduce a new parameter which divides concerned working boundary in number levels, which gives K+1 pre-localized node from each level. The reduction in storage capacity and complexity of PLD network localization plays a vital role. The ranging of Δ is also reduced to some level, which helps to reduce localization error.

Let τ be a storage matrix contains pre-localized nodes in a working boundary. with the help of step size, mid-point is moved all over the three dimensions of the networks and find out number of the pre-localized node. The dimension of storage matrix is 3×[τ×(K+1)].

(25) $$PreLoc_{cord}=\left[\begin{array}{cccc} x_{p\tau 0} & x_{p\tau 1} & \ldots & x_{p\tau k}\\ y_{p\tau 0} & y_{p\tau 1} & \ldots & x_{y\tau k}\\ z_{p\tau 0} & z_{p\tau 1} & \ldots & z_{p\tau k} \end{array}\right]$$

To find the actual maximum and minimum localization volume, coordinate points are calculated from stored pre-localized nodes on each axis.

(26) $$V_{localization}=x_{\zeta }\times y_{\zeta }\times z_{\zeta }$$

The pre-localized volume is our localized boundary which is obtained by:

(27) $$V_{pre\;Loc}=\int_{x_{min}}^{x_{max}}\int_{y_{min}}^{y_{max}}\int_{z_{min}}^{z_{max}}f\left(x,y,z\right)dxdydz.$$

Then the whole working boundary is divided on the *N*-clusters and each cluster has its maximum and minimum co-ordinate value from the storage matrix. Each cluster difference co-ordinate value is calculated as:

(28) $$\left(x_{n(\zeta )},y_{n(\zeta )},z_{n(\zeta )}\right)=\left[x_{n}|\begin{array}{c} max\\ min \end{array},y_{n}|\begin{array}{c} max\\ min \end{array},z_{n}|\begin{array}{c} max\\ min \end{array}\right];\;where\;n=1,2,.....N.$$

This will clearly gives us the form to calculate the position vector of *i*th localized nodes.

(29) $$\left(x_{L_{Pi}},y_{L_{Pi}},z_{L_{Pi}}\right)=\left(\frac{x_{n_{\zeta }}}{2},\frac{y_{n_{\zeta }}}{2},\frac{z_{n_{\zeta }}}{2}\right)+\left(x_{n_{min}},y_{n_{min}},z_{n_{min}}\right)$$

### 4.6. Relationship between Anchor, Parametric and Pre-Localized Nodes

Assuming that Δ is a constant value then the distance between two pre-locaized node will be Δ. To prove this let distance between two consective nodes is:

(30) $$P_{N}=\left\{P_{N-1}\right\}\pm \Delta$$

The above equation provides subdivision of earth surface where the difference between two points is Δ. The maximum increment and decrement on parametric points result in the same coordinate points on $M_{1}$. Then Equation (30) can be written as:

(31) $$P_{N}=M_{1}$$

(32) $$M_{1}=P_{1i}\pm \sum_{j=1}^{N-1}\Delta$$

If a working boundary is not regular then changing on control vertices in each iteration produce a different middle point. The change in center point is:

(33) $$\gamma ={\overset{´}{M}}_{j+1}-{\overset{´}{M}}_{j}$$

$$=\alpha_{k}{\overset{´}{M}}_{j}+\frac{\left(1-\alpha_{k}\right)}{k}\sum_{k=1}^{K}P_{jk}-\alpha_{k}{\overset{´}{M}}_{j-1}+\frac{\left(1-\alpha_{k}\right)}{k}\sum_{k=1}^{K}P_{(j-1)k}$$

$$=\alpha_{k}\left({\overset{´}{M}}_{j}-{\overset{´}{M}}_{j-1}\right)+\frac{\left(1-\alpha_{k}\right)}{K}\left(\sum_{k=1}^{K}P_{jk}-\sum_{k=1}^{K}P_{(j-1)k}\right)$$

$$=\alpha_{k}\left({\overset{´}{M}}_{j}-{\overset{´}{M}}_{j-1}\right)+\frac{\left(1-\alpha_{k}\right)}{k}\left(k\times \Delta \right)$$

(34) $$=\alpha_{k}\left({\overset{´}{M}}_{j}-{\overset{´}{M}}_{j-1}\right)+\left(1-\alpha_{k}\right)\left(\Delta \right)$$

If difference between two different central points is Δ then Equation (33) can be written as:

(35) γ=Δ

**Figure 5:**
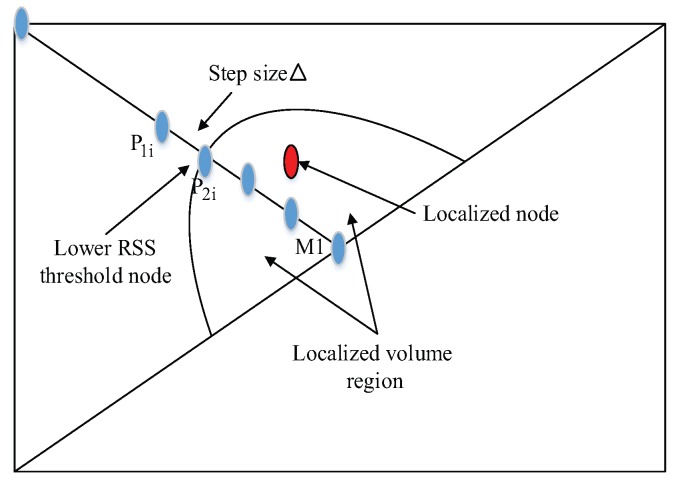
Localized volume region along with localized node.

Equation (29) shows that the points from Loop divisions are independent of the angle of deviation. Hence it’s described the the relationship between two parametric points. Those parametric points whose RSS value is less than the threshold are known as the pre-localized point. But our proposed system stores only first pre-localized point of each anchor node as shown in Figure 5.

**Figure 6:**
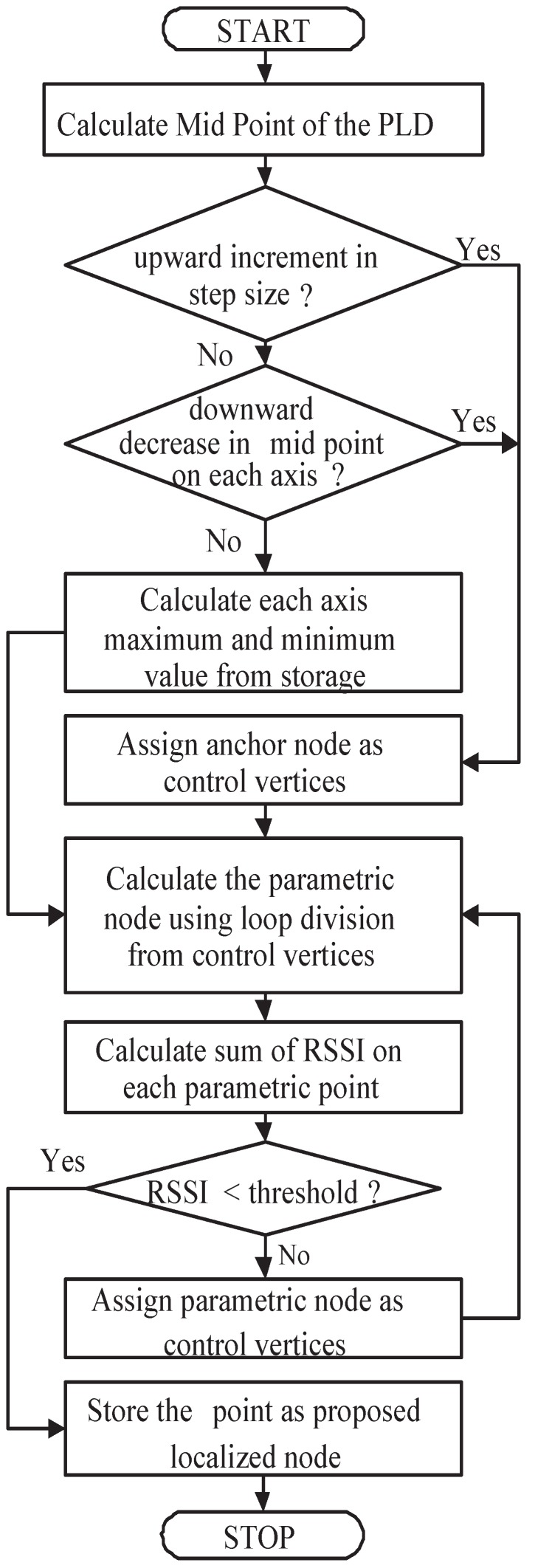
Flow diagram of PLD algorithm.

Equation (29) calculate the position of targeted nodes. The total number of actual points in 3D space under some specified unit volume is determined that satisfied the mathematical model we get:

(36) $$\left(\hat{x},\hat{y},\hat{z}\right)=[\left(K+1\right)P_{L}]\times \tau \times \frac{N}{k}$$

(37) $$Sum\left(LE\right)=\sum_{i=1}^{N}\sqrt{{\left(x_{i}-{\hat{x}}_{i}\right)}^{2}+{\left(y_{i}-{\hat{y}}_{i}\right)}^{2}+{\left(z_{i}-{\hat{z}}_{i}\right)}^{2}}$$

where LE represent localization error and $x_{i}$ is localized point and ${\hat{x}}_{i}$ is estimated point. The workflow diagram Figure 6 shows how PLD is implemented in WSNs.

## 5. Simulation Results

This section, provides a comprehensive evaluation of the PLD algorithm through simulation experiments on Matlab. Anchor nodes are randomly deployed within 100 m × 100 m × 100 m 3D area.

The number of anchor nodes in each simulation is set to 6 and, at each step, the location of anchor node is changed randomly. The simulation was run for 1000 iterations which make the deployment area to cover 6000 anchor nodes. Furthermore, taking a constant 80,000 m^3^ volume space for simulation on each axis. The total distance is then $d=\sqrt[{3}]{80000}=\;∼$95 m. From the lower bound the localization error is calculated from the following equation:

(38) $$l=\frac{0.955\sqrt{V}}{8\pi^{2}m\left(K+1\right)}$$

The number of localization points on PLD is directly proportional to the volume of Pre-localized nodes. As each node has localization error distance so we are interested in calculating mean error distance with constant sensing unit volume. Mean localization error (MLE) is calculated by the fraction of the number of nodes and sum of error distance. Table 3 shows the random deployment of anchor nodes that produces four localized point as the target node. Experiment shows the sum of localization error is 3.57 m and Mean localization error is 0.89 m.

For the same scenario, by selecting 10 different anchor nodes, we obtained different iterative values as shown in Table 4. From Table 4 the average error having 5 anchor nodes is 1.55 m, 1.58 m, 1.45 m, 1.26 m and with six anchor nodes is 1.43 m, 1.36 m, 1.12 m, 0.9 m. The simulation results of PLD shows, as the number of anchor nodes increases for a given environment, the localization error decreases. Furthermore, the obtained localization error is less when we choose the distributed anchor node positions properly.

In PLD, the localization error is affected by step size Δ. The value of Δ should not be higher in small networks. For a gived experimental area with five anchor nodes, PLD has a higher value of step size Δ as compared to PLD network with six anchor nodes. For the authenticity of PLD algorithm an average, minimum and maximum error is also recorded against A=5 and A=6 as shown in Table 5.

**Figure 7:**
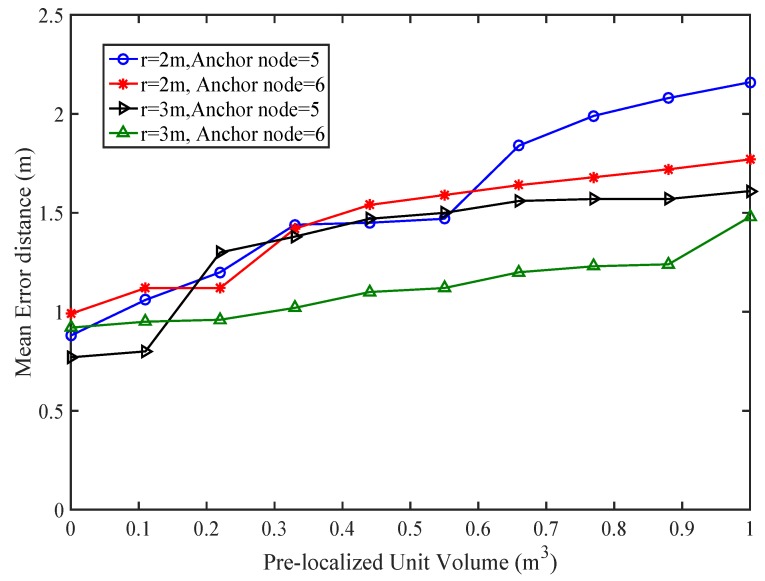
Mean error analysis with different volumes of PLD.

The accuracy with the higher number of anchor node can reduce the localization error. The simulation result is shown in Figure 7.

### 5.1. Effect of Rayleigh Fading

We have taken Rayleigh fading into account in studying the performance of PLD. Variation of signal amplitude over time and frequency gives unique characteristic of RSS cause by fading. To model that, the power samples have to be multiplied by a factor $r_{f}^{2}$ [38], where $r_{f}$ is a random variable accounting for the fading amplitude, which is modelled with a Rayleigh pdf as mentioned in Equation (21). To reflect the two main properties of radio irregularity, namely non-isotropic and countinuous variations, the path loss value is adjusted in Equation (29), based on the relationship $d=d_{0}+N\left(\mu ,\sigma \right)$ where, μ is mean and σ represents standard deviation.

**Figure 8:**
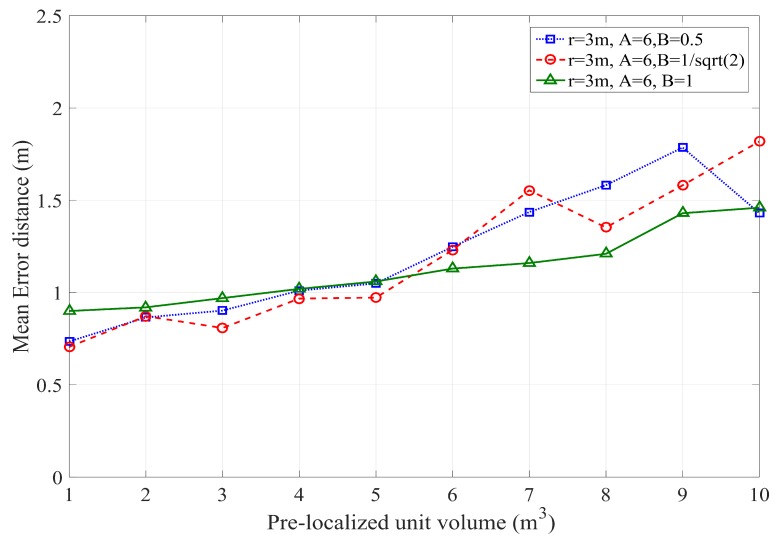
Effect of Multipath Fading on Localization Error.

As shown in Figure 8, Rayleigh fading is added to the RSSI to measure the multipath fading effect on localization. We have:

(39) $$RSSI=RSSI+20\mathrm{lg}(r_{f})$$

where $r_{f}$ is a multipath factor represents Rayleigh fading.

**Figure 9:**
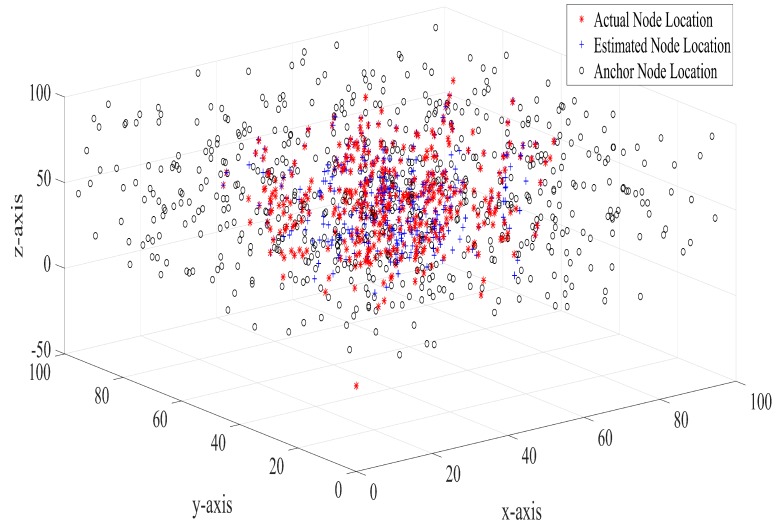
Location of anchor nodes, actual sensor nodes and estimated sensor nodes in 3D environment.

Figure 9 shows the anchor nodes, actual sensor nodes and estimated sensor deployment for 10 iterations of simulation. As can be seen in Figure 9, random deployment of anchor nodes results in the spread of anchor nodes across the deployment region. The distance between estimated sensor nodes and the actual sensor nodes are predominantly small.

**Figure 10:**
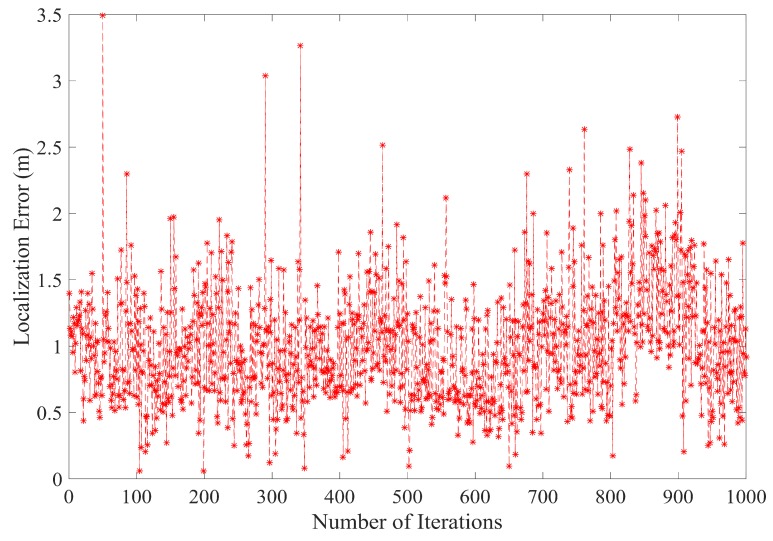
Average localization error after 1000 iterations.

Figure 10 shows the average localization error after 1000 iterations. As can be seen in Figure 10, the average localization is well within the limit. The average localization error, after 1000 iterations with A = 6 in each iterative step, is found to be between 0.9 m and 3.5 m. The main reason for this reduction in the localization error is that PLD algorithm utilizes all the ranges between a sensor node and anchor node. Since the number of anchor nodes locations used by a single sensor node is more than three, therefore, a better location estimate for sensor node position is obtained.

### 5.2. Localization Error under Varying Anchor Node Density

**Figure 11:**
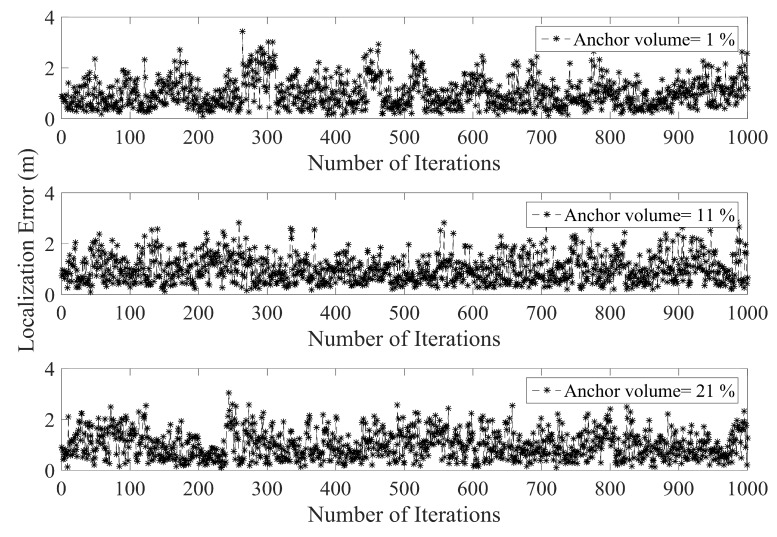
Localization error under different percentage of anchor node density.

**Figure 12:**
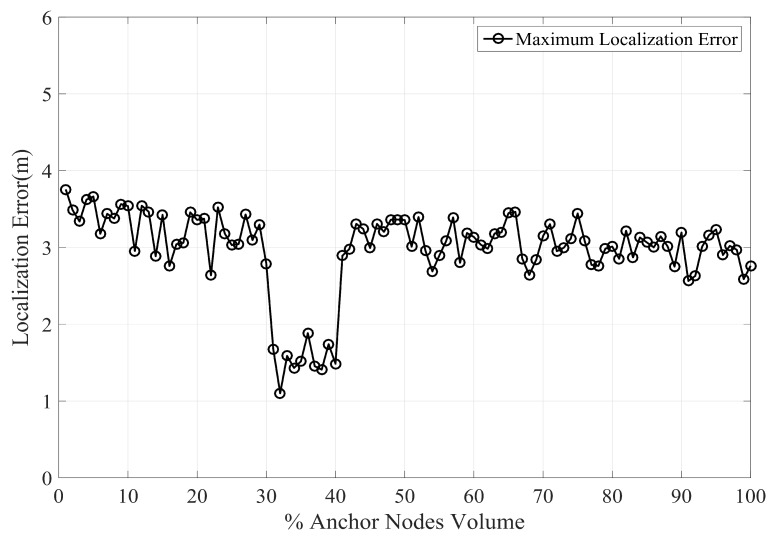
Localization error vs varying percentage of anchor node density.

By increasing the anchor node volume the error in PLD gradually decreases. Figure 11 shows the average localization using a different percentage of anchor nodes volume. With increasing anchor nodes density, the localization error is reduced. However, there is a certain limit beyond which the localization error ceases to reduce. This phenomenon has been shown in Figure 12, which indicate the maximum localization error with varying percentage of anchor node density. As can be seen in Figure 12, the maximum limit for anchor node density, resulting in reduce localization error, is within 29% to 30%.

**Figure 13:**
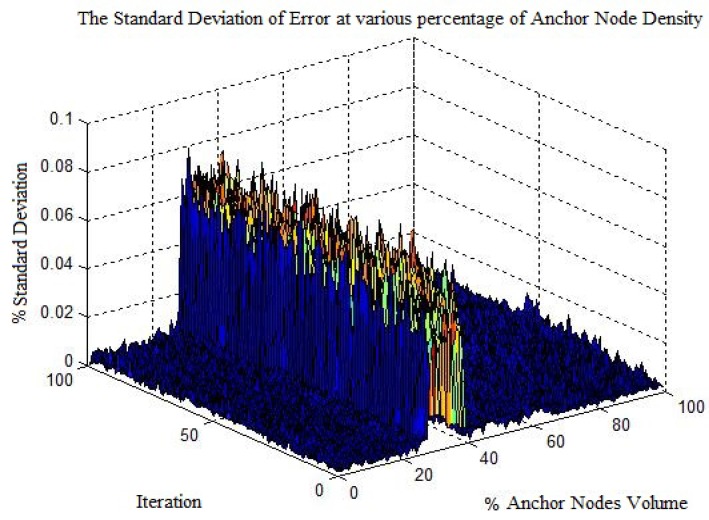
Percentage maximum standard deviation with varying anchor node volume.

Figure 13 shows the percentage standard deviation of localization error. It can be seen that at 29% to 30% we obtained a higher % standard deviation. The main reason for this high deviation is that the maximum localization error at the respective % anchor nodes density interval is reduced from the average localization error. Therefore one can assume an upper bound on the volume of anchor node for localization. The % standard deviation is calculated by:

(40) $$\%SD=\frac{\sqrt{E{\left(D_{i}-\mu \right)}^{2}}\times 100}{\sum D_{i}}$$

where

(41) $$D_{i}=\sqrt{{\left(P_{LP_{i}}-P_{eP_{i}}\right)}^{2}}$$

### 5.3. Comparison with Existing Methods

**Figure 14:**
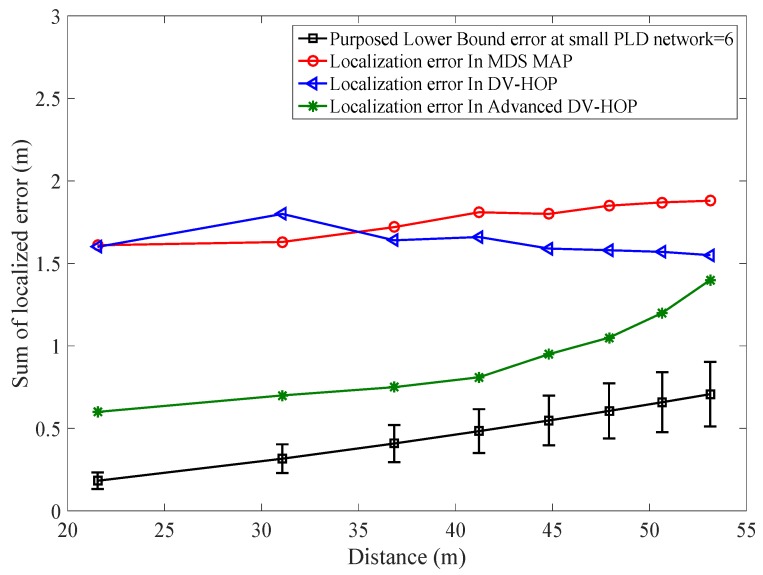
Comparison of lower bounds PLD network error to existing systems.

The simulation was run for 100 times to obtain an average localization error. PLD shows superior performance rather than APIT [31], AD-Hoc [32], and MDS-Map [33] schemes as shown in Figure 14. PLD produce number of localization point depends upon radial distance. RSSI of −40 dBm is settled as a threshold value for producing localization points.

**Figure 15:**
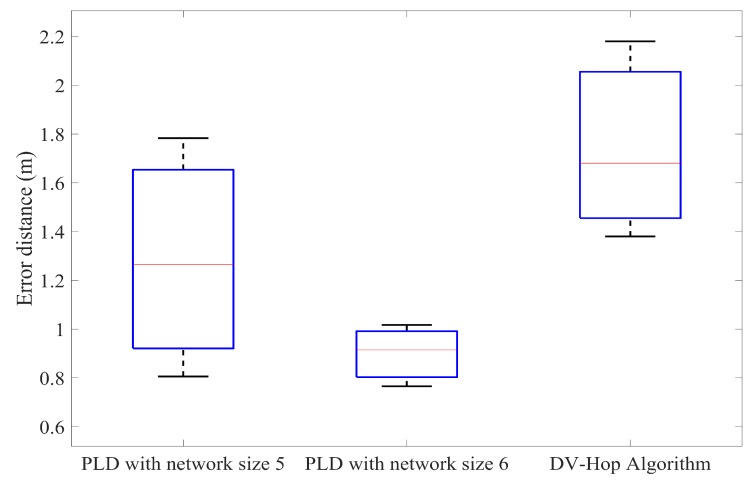
Comparison of the average position error of PLD with DV-Hop at 20% anchor nodes.

**Figure 16:**
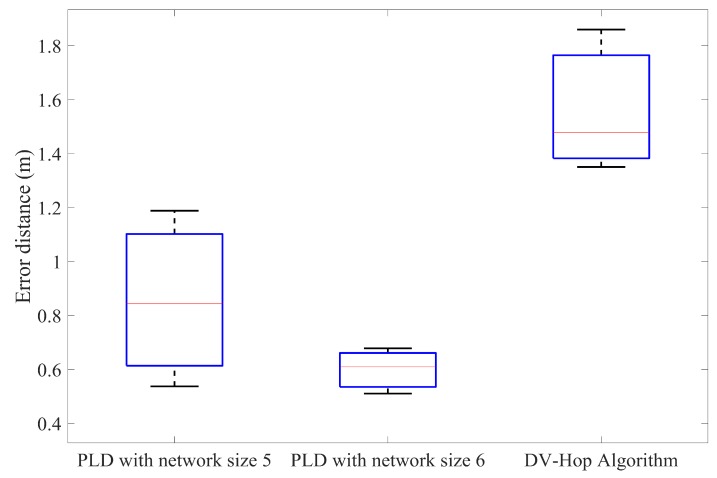
Comparison of the average position error of PLD with DV-Hop at 25% anchor nodes.

**Figure 17:**
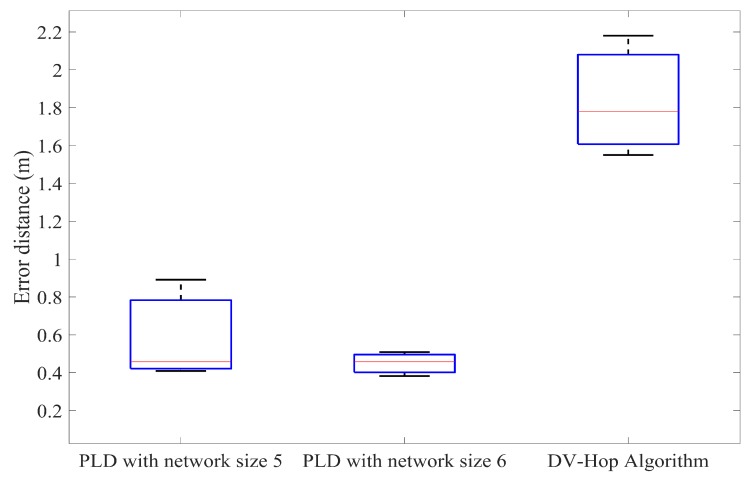
Comparison of the average position error of PLD with DV-Hop at 30% anchor nodes.

Furthermore, the PLD has been simulated with different anchor node percentage and compared with the DV-Hop method. In each of the iteration, by increasing the number of anchor nodes percentage to achieve accuracy. Figure 15, Figure 16 and Figure 17 illustrate this in the form of box plot.

### 5.4. Accuracy Analysis of PLD Algorithm

**Figure 18:**
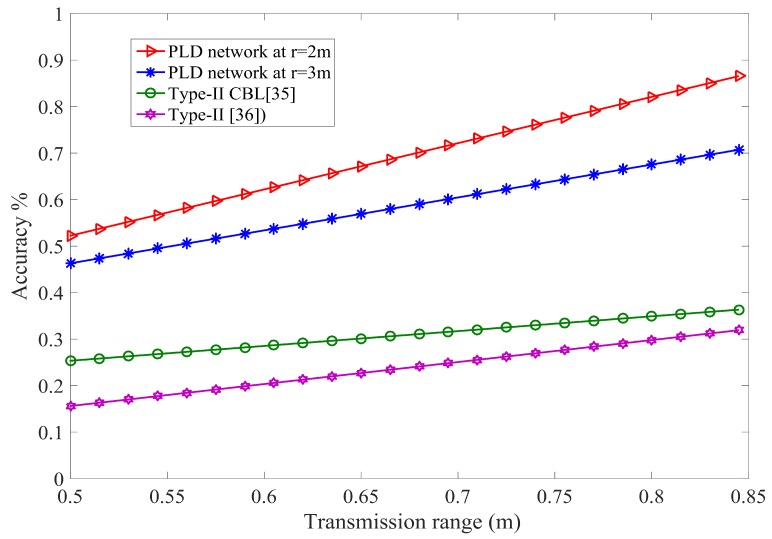
Impact of transmission range and localization accuracy of PLD with different network size.

The expected localization error in PLD utilizes equal probability at each node over a deployment region. Because all localization nodes follow same uniform distribution of anchor nodes in 3D space. The cumulative distribution function (CDF) of the error distance can be defined as e(r)=P(D<r) where probability density function PDF is calculated under unit volume. If the sensor nodes are uniformly distributed over a region *R*, then PDF function of volume *V* is

(42) $$f\left(x,y,z\right)=\frac{1}{V_{R}}$$

(43) $$\varrho =\frac{2\times Error\;distance\;on\;each\;axis}{Distance_{N⟶N}}=\left\{\frac{1-U}{2},\frac{1+U}{2}\right\}$$

where ϱ is unit transmission ratio. The unit sensing radius of actually localized node plays a vital role in the accuracy of PLD network. r=2 m and r=3 m are chosen for accuracy analysis. The transmission range is calculated under radius of sensing between two localized nodes. The transmission range of r=2 and r=3 is 4 m and 6 m respectively. The accuracy of PLD network is similar to [39], however, PLD networks operate on the volume basis. The minimum worst case accuracy of PLD network is 0.653 and 0.681 for PLD network size 5 and size 6 respectively at transmission range of d=0.76346. It gives comparatively higher tolerance level than 0.2887 in [39] and 0.28286 in [40], which is shown in Figure 18.

To further measure the accuracy, let us consider a huge network region *R* is divided in to several non overlapped networks $R=\left\{R_{1},R_{2},\ldots R_{k}\right\}$ with volume *V* i.e., $V=\left\{v_{1},v_{2},\ldots v_{k}\right\}$. Any single sensor node ρ of PLD network has localization error $l_{e}=\left(x,y,z\right)$ lies in the real position at sub region $R_{i}$.

(44) $$\rho \left(V_{i}\right)=\frac{v_{i}}{V}\;and\;\sum_{i=1}^{k}\rho \left(V_{i}\right)=1$$

If $l_{e}\left(R_{i}\right)$ be the expected localization error of ρ(x,y,z) lying in a uniform distribution, then the sum of error $E[l_{e}]$ is being calculated by:

(45) $$E\left[l_{e}\right]=\sum_{i=1}^{k}\rho \left(V_{i}\right)l_{e}\left(R_{i}\right)$$

where $l_{e}\left(R_{i}\right)$ is derived from Equation (30).

(46) $$l_{e}\left(R_{i}\right)=\frac{1}{v_{i}}\int \int \int_{R_{i}}\sqrt[{3}]{X_{i(\zeta )}\times Y_{i(\zeta )}\times Z_{i(\zeta )}}dxdydz$$

**Figure 19:**
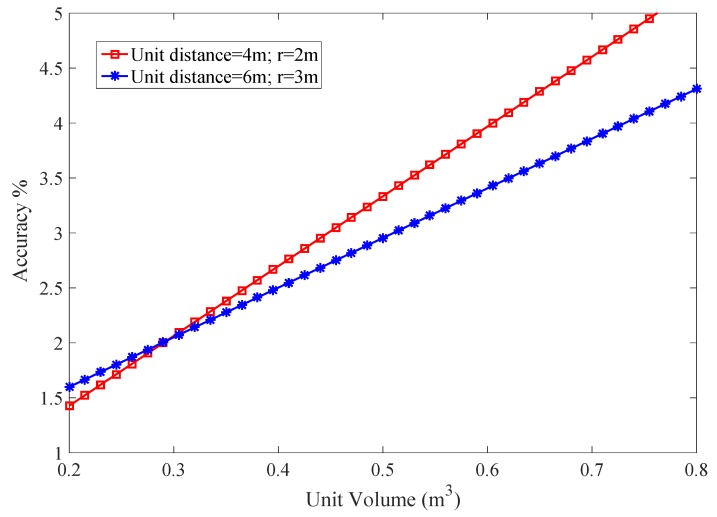
Accuracy of PLD network with different volume of PLD network.

Substitute Equation (46) into Equation (45):

(47) $$E\left[l_{e}\right]=\sum_{i=1}^{k}v_{i}l_{e}\left(R_{i}\right)$$

where ζ is a difference between the coordinates. Now working volume is transferred to the rectangular 3D space of PLD region. This shows the accuracy and justification of the PLD algorithm. The PDF is varied according to variation in volume. Volume step size function of varied volume is taken as constant that estimate coverage volume each time. The localization accuracy with the unit transmission is shown in Figure 19.

**Figure 20:**
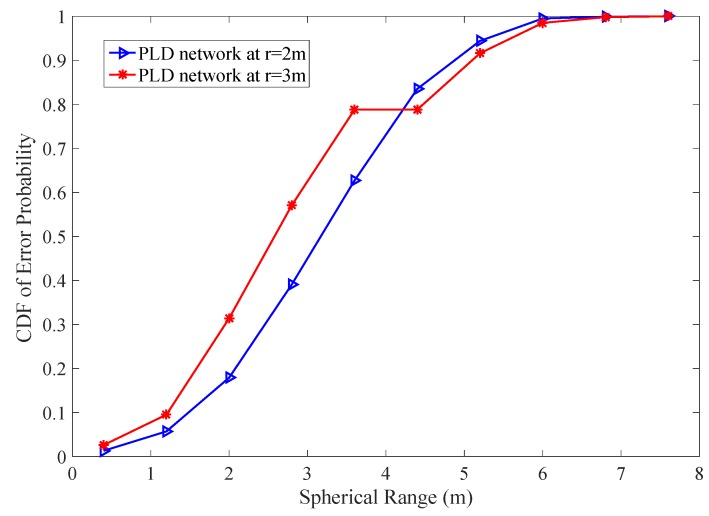
Cumulative error probability in PLD network with r=2 m and r=3 m.

To calculate the probability of PLD, we perform 1000 random experiments where each experiment have 10 trial events in 10 m spherical distance. We are interested in finding out localization probability at two unit sensing radius r=2 m and r=3 m, respectively. The minimum radius taken from [40] which has probability = 1. Our experiment has localization probability 0.5 and 0.333 respectively with 10,000 different probability values for each PLD network. The cumulative sum of localization probability shown in Figure 20. The error probability lies beyond the range of working boundary. The lower sensing radius gives less error probability in PLD network. The trade-off between unit sensing radius and radio coverage is found in PLD network.

### 5.5. Effect of Anchor Node Position Error

**Figure 21:**
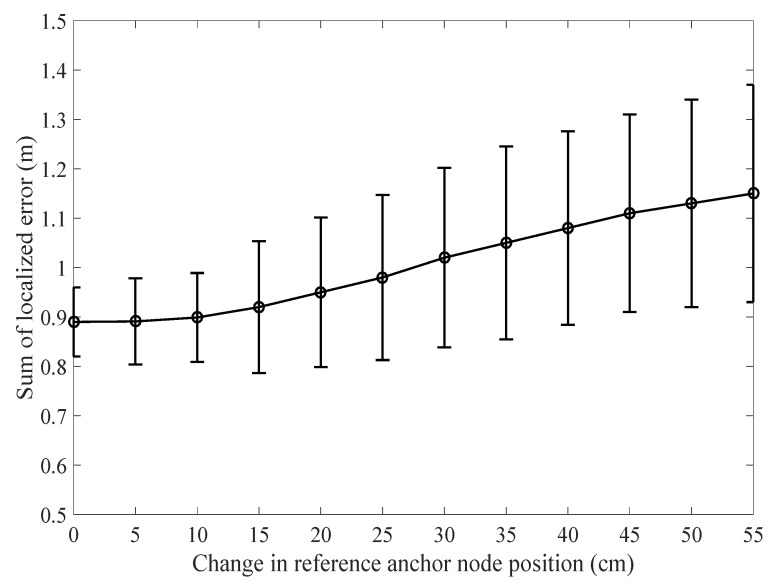
Influence of reference anchor node position vs localization error.

In the literature, much attention has been paid to localization accuracy and computational effort, while the importance of intelligent anchor node placement is often recognized, but not discussed in detail. In [41,42], anchor nodes were randomly deployed. In [41], authors mentioned that the co-linear set of anchors “represents a rather unlucky selection” without supporting evidence. For PLD, good anchor node placement is important to form a working boundary. As we know, the localization error is the difference in distance between the actual node and the estimated node position. In the context of global localization, which reflects how accurately the calculated global coordinates are matched with the actual coordinates. To achieve that, anchor nodes should be deployed in a way so that it can form a correct localization boundary. Hereafter, we investigate how the anchor node position can affect the localization accuracy. This work only describes the effect of anchor node position effect on overall PLD operations. Other metrics for anchor node influences like anchor node localization error, network area coverage, and anchor node triangle (deployment height and position) are left for future study. We could also explore how to avoid the worst anchor node placement and use of correct topology. Now, we can check the localization error by changing the location of anchor nodes in centimeter on each side and gradually increase the distance. As shown in Figure 21, we can see that the localization error gradually increases as the shift of anchor node position increases.

### 5.6. Time Complexity and Lower Bound Derivations

The computational complexity of the network is relative to the time consumption of the network. Let PLD network system has minimum $\frac{N}{k}$ to maximum *N* PLD networks in a huge distribution of network.

**Figure 22:**
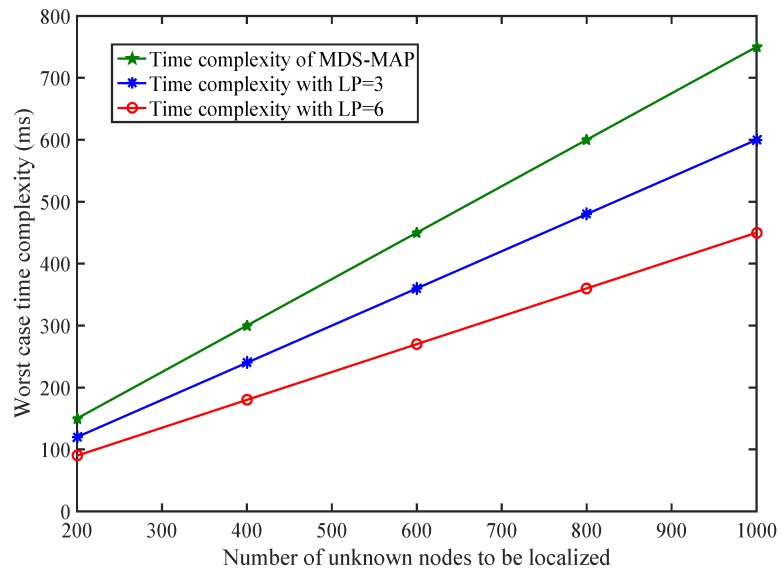
Complexity comparison between PLD and multi-dimensional scaling (MDS)-MAP.

**Figure 23:**
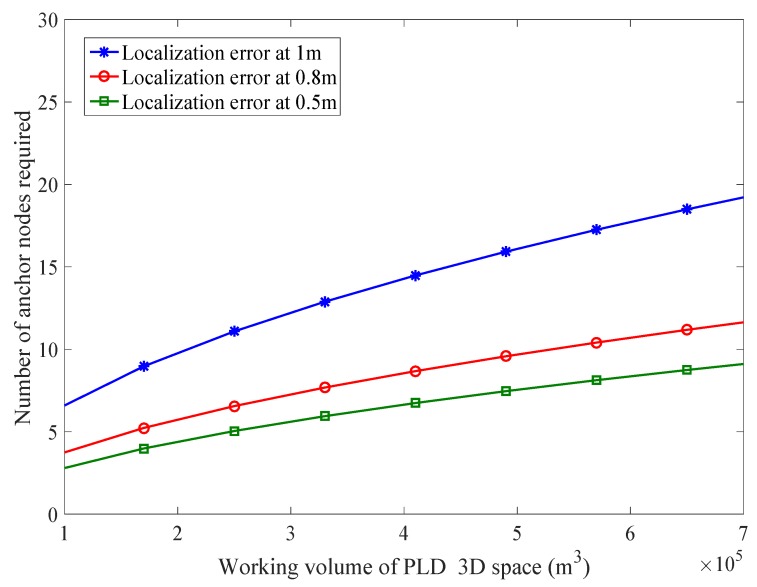
Number of anchor nodes required and their corresponding lower bounds.

The complexity is reduced up to 75%. However, if we have N=7 the complexity is completely removed. Complexity is being compared with MDS-MAP as shown in Figure 22. Each PLD network estimates the number of simultaneous localization points ξ. The number of known anchor nodes in our experiment space are $\left(\frac{N}{K}\times \xi \right)\leq N_{PLDnetwork}\times \xi \leq N\times \xi$ that satisfy our formulated model. Consider a WSN having 400 sensor nodes with 50 anchor nodes. The number of unknown sensor nodes to be localized is 350. If our system calculates five simultaneous anchor nodes, then N=5. So the number of known nodes is $\left(50+10\times 5\right)=100\leq \left(50+50\times PLD_{network}\times 5\right)\leq \left(50+50\times 5\right)=300$. The requirement of number of anchor nodes along with different volume of PLD is shown in Figure 23.

For computing lower bound derivations let le(C) be the value of $l_{e}\left(R_{i}\right)$. For regular unit shape $sh_{R_{i}}$ defined as $e_{sh_{R_{i}}}$ is derived by

(48) $$e_{sh_{R_{i}}}=\frac{le(R_{i})}{le(C)}=0.9554$$

By dividing each deployment region by unit sphere gives a scaler value

(49) $$e_{sh_{R_{i}}}=\frac{le(R_{i})}{m\times le(C)}=0.9554$$

where $m=\frac{v_{i}}{c}$. By putting above value in sum of error equation we get

(50) $$E\left[le\right]=\frac{1}{V}\sum_{i=1}^{k}\frac{v_{i}}{C}e_{sh_{r_{i}}}le\left(C\right)$$

We can obtain the minimum value of sphere volume as $\frac{1}{8\pi^{2}}$ . The worst case error can be calculated through $l=\frac{0.955\sqrt{V}}{8\pi^{2}m\left(K+1\right)}$ where *V* is the numerical value and $k=\frac{0.955\sqrt{V}}{8ml\pi^{2}}-1$.

**Figure 24:**
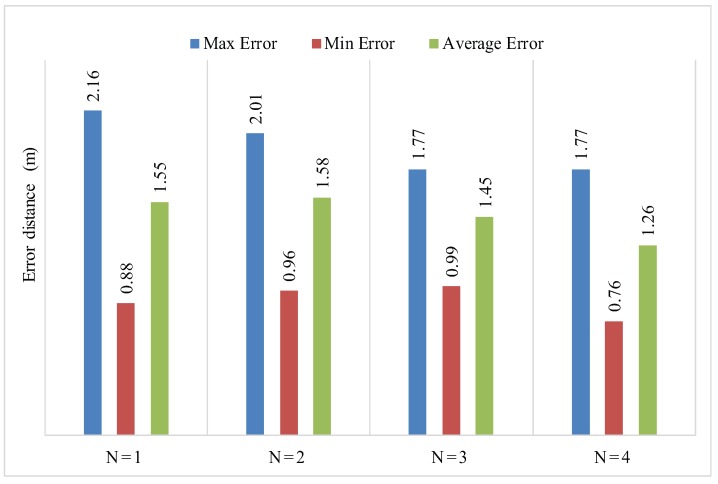
Localization error distance of PLD with A = 5.

**Figure 25:**
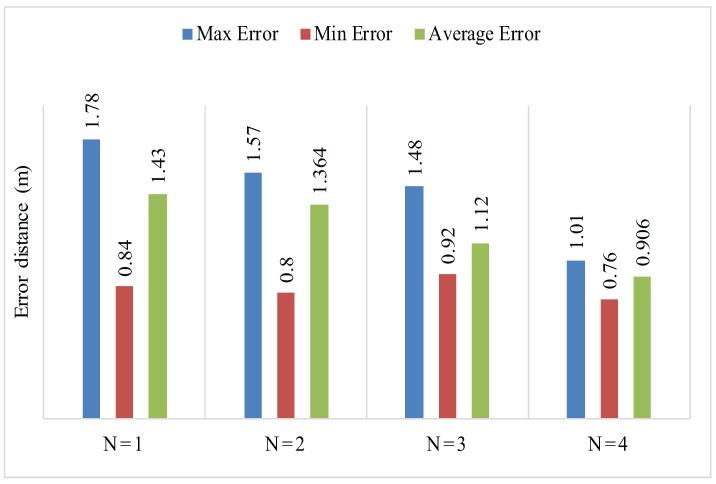
Localization error distance of PLD with A = 6.

**Figure 26:**
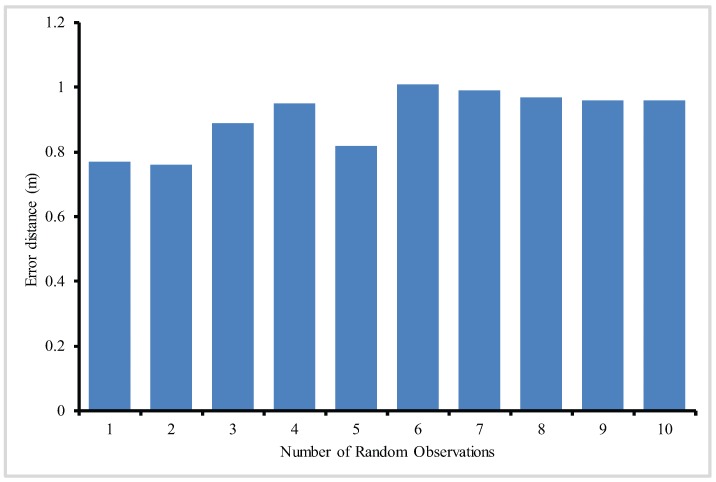
Random experiment of localization error of PLD with six anchor nodes in each cluster.

**Figure 27:**
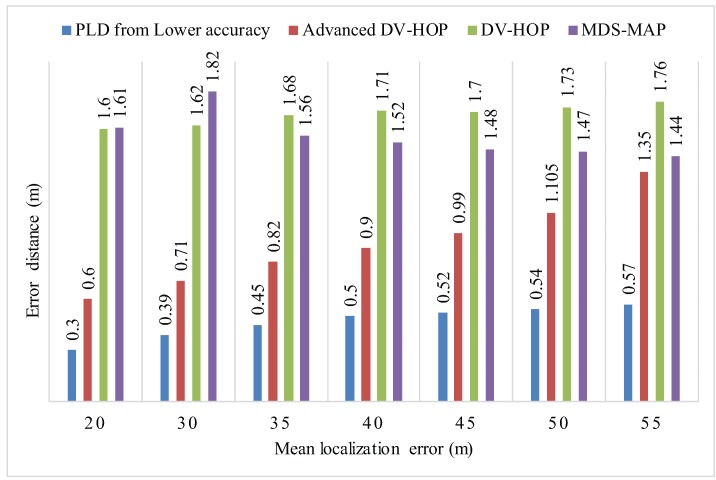
Mean localization error of PLD, DV-Hop, Advanced DV-Hop and MDS-MAP.

Figure 24, shows the mean localization error of PLD algorithm. Figure 25 and Figure 26, describe the PLD localization error with each network cluster having 5 and 6 anchor nodes respectively. Figure 27 shows the different observations of PLD simulations. For this, we used lower bounding error that greatly reduced the localization error which is superior to existing system. Figure 27 shows the mean localization error between DV-Hop, MDS-MAP, and PLD.

## 6. Conclusions

Node localization plays a vital role in improving computational efforts in wireless sensor networks. Many researchers have proposed different localization techniques for 2D based sensor network. However, most of them are based on the assumptions of accurate synchronization between sensor nodes, which can be difficult or sometimes impossible to achieve in certain environment. This paper proposed a novel 3D localization algorithm based on the well-known parametric Loop division algorithm, which is free from node synchronization and thus only required to determine the mid-point to form a working boundary. PLD is able to divide the whole region into several networks, which can overcome the computational overhead and communication cost.

PLD is capable of finding its own localized node within its working boundary. At first, reference points are considered to produce mid-points, parametric points and step size, which helps the iterative control to be transferred to inner parametric points. This enables PLD to work in different networks, within the working boundary. At each reference point, sum of RSSI value is computed for pre-localized nodes, compared to a threshold value, and stored in a storage matrix. Furthermore, the localization volume is obtained with maximum and minimum coordinates, stored in a storage matrix. Finally, we can estimate the position of the node by performing centroid localization using the information in the storage matrix. Through simulation comparisons, our proposed scheme outperforms existing schemes. PLD achieves an error of 0.89 m which is far better than most of well-known existing schemes like APIT, DV-Hop, and MDS-MAP. The simulation results showed that the localization accuracy is improved as the number of anchor nodes increased. Furthermore, the position change of an anchor node will only gracefully affect the localization accuracy.

However, there are still some room for further studies, such as the impact of anchor localization error. It is also worthy studying how to adopt mobile anchor nodes to further improve the localization accuracy. In addition, energy consumption of PLD localization should be investigated and the trade-off between localization accuracy and energy consumption can be identified. Finally, we shall study how to integrate PLD localization with other techniques.

## Figures and Tables

**Table 1:** Indoor positioning technologies.

Technology	Typical Accuracy	Typical Coverage (m)	Measurement Technique
**Camera**	0.1 mm∼1 dm	1∼10	Angle measurements from images
**Infrared**	1 cm∼1 dm	1∼5	Active beacons
**Sound**	2 cm	2∼10	Time of Arrival (ToA)
**Wi-Fi**	1 m	20∼50	Fingerprinting
**RFID**	1 dm∼1 m	1∼50	Fingerprinting, proximity detection
**UWB**	1 cm∼1 m	1∼50	ToA, body reflection
**Pseudolites**	1 cm∼1 dm	10∼1000	Carrier phase ranging
**Magnetic systems**	1 mm∼1 cm	1∼20	Fingerprinting and ranging technique
**Zigbee**	1 m	30∼60	Centroid based techniques

**Table 2:** List of key notations.

Notation	Explanation
$M_{i}$	Mid-points of each PLD network
$A_{i}$	*i*th anchor node
$P_{i}$	*i*th parametric points produced after each iteration
$v_{i}$	Volume of *i*th parametric looped network
$k_{i}$	Non overlapped PLD networks
$D_{N\to N}$	Distance matrix from a sensor node $N_{i}$ to all other sensors in a network
$D_{A\to N}$	Distance matrix from a anchor node $A_{i}$ to all other sensors in a network
φ	Targetted node in each $k_{i}$ network
η	Number of generated anchor nodes in $k_{i}$ network
Δ	Step size in PLD network
α	Parametric function of PLD network
γ	Representation of change in center point
ξ	Working boundary
$\hat{x},\hat{y},\hat{z}$	Cartesian coordinates of estimated node position.

**Table 3:** Localization error of four nodes in each PLD network.

x̂	ŷ	ẑ	x	y	z	Error in (m)
14.47	7.66	14.11	15.90	8.20	15.27	1.91
15.54	9.93	14.90	15.54	9.93	14.90	1.53
15.73	10.65	15.25	15.79	10.63	15.27	0.05
16.93	11.85	16.45	16.94	11.85	16.15	0.08

**Table 4:** Mean error of 10 different trials of PLD network with r=3 m.

N = 1		N = 2		N = 3		N = 4	
A = 5	A = 6	A = 5	A = 6	A = 5	A = 6	A = 5	A = 6
1.06	0.84	2.01	1.5	1.42	1.48	1.77	0.77
1.2	1.08	1.99	1.36	1.77	1.08	1.22	0.76
1.44	1.62	1.93	1.38	1.68	1.10	0.91	0.89
1.45	1.60	1.57	1.47	1.64	1.12	1.52	0.95
1.84	1.78	1.30	1.57	1.72	1.23	1.44	0.82
2.16	1.75	1.69	1.56	1.12	1.24	1.41	1.01
1.99	1.66	0.96	1.57	1.54	1.20	0.76	0.99
2.08	1.57	1.25	1.61	1.12	0.95	0.78	0.97
1.47	1.26	1.73	0.77	0.99	0.96	1.42	0.96
0.88	1.18	1.43	0.80	1.59	0.92	1.43	0.96

**Table 5:** Avg, Max, and Min localization error at each PLD network with A = 5 and A = 6.

Number of Localization Points	*e_avg_*		*e_max_*		*e_min_*	
	A = 5	A = 6	A = 5	A = 6	A = 5	A = 6
N = 1	1.55	1.43	2016	1.78	1.28	0.84
N = 2	1.58	1.364	2.01	1.61	1.05	0.77
N = 3	1.45	1.128	1.77	1.48	0.78	0.92
N = 4	1.265	0.908	1.77	1.01	1.01	0.76

## Appendix A

Let the parametric points be

(A1) $$P_{ik}=\left\{P_{11},P_{12},....,P_{1k}\right\}.$$

New center points are calculated by using parametric factor. The new center points which is less dependent with irregular distribution of anchor node, can be calculated as:

(A2) $${\overset{´}{M}}_{1}=\alpha_{k}M_{1}+\frac{\left(1-\alpha_{k}\right)}{k}\sum_{k=1}^{K}P_{ik}$$

where $\alpha_{k}$ represent parametric function of PLD network obtained from [40] whose value is constant if anchor node has regular distribution. Due to symmetry, we can also write this for anchor node distribution

(A3) $${\overset{´}{M}}_{1}=\alpha_{k}M_{1}+\frac{\left(1-\alpha_{k}\right)}{k}\sum_{k=1}^{k}A_{ik}$$

(A4) $${\overset{´}{M}}_{1}=\alpha_{k}M_{1}+\left(1-\alpha_{k}\right)\frac{\sum_{k=1}^{K}\left(A_{ik}\right)}{k}$$

(A5) $${\overset{´}{M}}_{1}=\alpha_{k}M_{1}+\left(1-\alpha_{k}\right)M_{1}$$

i.e., For regular distribution the centroid of points lies in centre i.e., ${\overset{´}{M}}_{1}=M_{1}$

In case of irregular distribution the value of $\alpha_{k}$ lies between 0.5 to 0.75. Hence,

(A6) $$\alpha_{k}=\frac{3}{8}+{\left(\frac{3}{8}+\frac{1}{4}\cos\frac{2\pi }{k}\right)}^{2}$$

To compute radio irregularity we take two different values of $\alpha_{k}$. One is 0.5 for assuming center value and 0.75 for anchor nodes. $\alpha_{k}$ has direct effect on cosine angle that is between two anchor nodes from the center points in triangulation. Suppose the difference in angle is (0∼90)°, then the value of $\alpha_{k}$ ranges from 0.516 to 0.765.

$M_{1}$ has a parametric factor $\alpha_{m}=\alpha_{k}=\frac{2\pi }{k}$ and $\alpha_{p}=\alpha_{k}=\frac{\alpha_{1},\alpha_{2},....,\alpha_{p}}{k}$. So the next mid-point is

(A7) $${\overset{´}{M}}_{1}=\alpha_{m}M_{1}+\left(1-\alpha_{p}\right)M_{1}$$

(A8) $${\overset{´}{M}}_{1}=\left(1+\alpha_{m}-\alpha_{p}\right)M_{1}$$

The change in parametric value shifts the assuming center to the exact center.

## Appendix B

For perfect mathematical modelling we, assume that anchor nodes are regularly distributed, the sum of acute angle making with center is equal to 360°.

If k=5 A=5, cosθ has value of 0.3090 and θ = 72°. And the value of $\alpha_{k}=0.5795$.

If k=6 A=6, cosθ has value of 0.5 and θ = 60°. And the value of $\alpha_{k}=0.625$.

If irregular anchor node distribution occurs, then we consider irregular distribution of angle between anchor nodes. The angle effect on parametric factor is significant only when it has significant difference between angles.

If angle varies by 10° at k=5 then the value of θ lies between θ = (67∼77°) and the value of $\alpha_{k}$ = (0.5984∼0.5610). Hence localization error = 0.0374. Similarly, if angle varies by 10° at k=6 then the value of θ lies between θ = (55∼65°) and the value of $\alpha_{k}$ = 0.6437∼0.6060. Hence localization error = 0.0377. This shows that anchor node irregularity produce some considerable error but we minimize it. The minimization occur because we calculate only midpoint of each iteration by using this parametric function. The difference of shifting is greatly minimized by:

(A9) $${\overset{´}{M}}_{1}=\left(1+\alpha_{p}-\alpha_{m}\right)M_{1}$$

with the localization error of 0.0377 in a parametric factor equation, we get; ${\overset{´}{M}}_{1}=\left(1-0.0377\right)M_{1}=0.9623M_{1}$.

From the numeric parametric analysis, it is clearly seen that less angle gives higher parametric value $\alpha_{p}$ and higher angle gives lower parametric constant $\alpha_{m}$.
